# Removing unwanted variation from large-scale RNA sequencing data with PRPS

**DOI:** 10.1038/s41587-022-01440-w

**Published:** 2022-09-15

**Authors:** Ramyar Molania, Momeneh Foroutan, Johann A. Gagnon-Bartsch, Luke C. Gandolfo, Aryan Jain, Abhishek Sinha, Gavriel Olshansky, Alexander Dobrovic, Anthony T. Papenfuss, Terence P. Speed

**Affiliations:** 1 Walter and Eliza Hall Institute of Medical Research, Parkville, Victoria Australia; 2 Department of Medical Biology, The University of Melbourne, Melbourne, Victoria Australia; 3 Biomedicine Discovery Institute and the Department of Biochemistry and Molecular Biology, Monash University, Clayton, Victoria Australia; 4 Department of Statistics, University of Michigan, Ann Arbor, Ann Arbor, MI USA; 5 School of Mathematics and Statistics, The University of Melbourne, Melbourne, Victoria Australia; 6 Department of Economics and Statistics, Monash University, Melbourne, Victoria Australia; 7 Metabolomics Laboratory, Baker Heart and Diabetes Institute, Melbourne, Victoria Australia; 8 Baker Department of Cardiometabolic Health, The University of Melbourne, Melbourne, Victoria Australia; 9 Department of Surgery, The University of Melbourne, Austin Health, Heidelberg, Victoria Australia; 10 Peter MacCallum Cancer Centre, Melbourne, VIC Australia; 11 Sir Peter MacCallum Department of Oncology, The University of Melbourne, Melbourne, Victoria Australia

**Keywords:** Statistical methods, Cancer genomics

## Abstract

Accurate identification and effective removal of unwanted variation is essential to derive meaningful biological results from RNA sequencing (RNA-seq) data, especially when the data come from large and complex studies. Using RNA-seq data from The Cancer Genome Atlas (TCGA), we examined several sources of unwanted variation and demonstrate here how these can significantly compromise various downstream analyses, including cancer subtype identification, association between gene expression and survival outcomes and gene co-expression analysis. We propose a strategy, called pseudo-replicates of pseudo-samples (PRPS), for deploying our recently developed normalization method, called removing unwanted variation III (RUV-III), to remove the variation caused by library size, tumor purity and batch effects in TCGA RNA-seq data. We illustrate the value of our approach by comparing it to the standard TCGA normalizations on several TCGA RNA-seq datasets. RUV-III with PRPS can be used to integrate and normalize other large transcriptomic datasets coming from multiple laboratories or platforms.

## Abstract (web summary)

Cancer RNA-seq data are normalized with respect to library size, tumor purity and batch effects.

## Main

An essential step of RNA sequencing (RNA-seq) data analysis is normalization, whereby different sources of unwanted variation are removed to make gene expression measurements comparable within and between samples^1–4^. In cancer RNA-seq data, within-sample normalization should adjust for gene length, GC content and cellular compositions, whereas between-sample normalization should remove the impact of library size, tumor purity and batch effects on the data. Efficient removal of such variation from RNA-seq data is still a challenge. This variation can introduce artifactual or obscure true biological signals in the data and, consequently, lead to false or missed discoveries, resulting in misleading biological conclusions^1,5–8^.

Most RNA-seq normalizations adjust for library size variation using global scaling factors calculated based on either total counts or other statistical features of the raw count data, such as their upper quartiles^3,9,10^. These normalizations simply divide all gene counts in each sample by a single scale factor. The implicit assumption underlying such methods is that all the gene-level counts are proportional to the scale factors and that it should be adequate to adjust them for library size in this way across samples. A current challenge for RNA-seq normalizations arises when the counts for a reasonable proportion of genes cannot be properly adjusted for library size by the use of a single scale factor, regardless of how it is computed. The bias between gene-level counts and library size has been discussed in single-cell RNA sequencing data^11,12^; however, this has not been recognized in RNA-seq data.

Tumor purity—that is, the proportion of cancer cells in solid tumor tissues—is another major source of variation in cancer RNA-seq data. This variation has been viewed as an intrinsic characteristic of tumor samples and has been linked to several clinical outcomes in patients with various cancer types^13–16^. Tumor purity could be considered as a source of unwanted variation in studies whose aims are restricted to tumor-specific expression. Variation in tumor purity can affect comparisons of a gene’s expression within and between samples, which can compromise downstream analyses in cancer RNA-seq studies^17–19^. Current RNA-seq normalizations and batch correction methods are unable to remove this kind of variation from the data. Adjusting counts for tumor purity variation using regression models risks removing biological signal if that signal is confounded with purity.

Batch effects are obvious sources of unwanted variation in large RNA-seq studies, where samples are necessarily processed across a range of conditions—for example, chemistry, protocol and facility. Most batch correction methods are based on linear regression. For individual gene expression, they fit a linear model with blocking terms for batch. Then, the coefficient for each blocking term is set to zero, and the corrected expression values are computed from the residuals^20–22^. An implicit assumption underlying such methods is that the biological populations are evenly distributed within each batch—that is, that there is no association between batch and biological condition. However, if there is such an association (due to confounding), then correcting gene expression counts for batch effects using these methods risks removing biological signal along with the batch effects. Furthermore, batch effects usually influence subsets of genes in different ways^6,8^; sample-wise normalization, including normalizations that rely on global scaling factors, generally fail to remove this variation from the data.

We previously developed a normalization method, called removing unwanted variation III (RUV-III), for gene expression studies with technical replicates^8^. The RUV-III method is a linear model through which the presence and impact of known and unknown unwanted factors can be inferred via technical replicates and negative control genes. However, RUV-III has two limitations. First, it is not designed to be used effectively in situations where technical replicates are not available or well-distributed across the sources of unwanted variation. Second, because a sample’s tumor purity will be essentially the same across all of its technical replicates, the original RUV-III is unable to estimate and remove this kind of variation using standard technical replicates.

Here we propose an approach, called pseudo-replicates of pseudo-samples (PRPS), to deploy RUV-III to efficiently remove the impact of library size, tumor purity and batch effects from RNA-seq data. The PRPS approach overcomes the limitations of RUV-III in situations where suitable technical replicates are not available or where variation due to tumor purity is to be removed from cancer RNA-seq data. To use RUV-III with PRPS, we first need to identify the sources of unwanted variation and major expression-based biological populations in the data. We then create pseudo-samples, which are in silico samples derived from small groups of samples that are roughly homogeneous with respect to unwanted variation and biology. Two or more pseudo-samples with the same biology will be regarded as a pseudo-replicate set. The gene expression differences between such pseudo-samples will largely be unwanted variation. RUV-III makes use of such differences, together with negative control genes, to estimate and remove unwanted variation from the data.

We make use of three RNA-seq datasets from The Cancer Genome Atlas (TCGA) studies to show that RUV-III with PRPS can effectively remove library size, tumor purity and batch effects and lead to meaningful biological results that are not compromised by this kind of variation. We will demonstrate that RUV-III with PRPS can be used to normalize multiple RNA-seq studies. We also present comprehensive strategies for revealing unwanted variation in large-scale RNA-seq studies, such as those of the TCGA project.

## Results

### TCGA RNA-seq datasets

The TCGA Research Network generated RNA-seq data from ~11,000 tumor and normal sample tissues obtained from 33 cancer types. To understand some potential sources of unwanted variation, fresh-frozen tissue samples were collected from tissue source sites (TSSs), allocated to 96-well sequencing plates (hereafter called plates) and processed at various times (Supplementary Table 1). Some TCGA RNA-seq datasets, such as uveal melanoma and kidney chromophobe, were generated using a single plate. In general, plates are completely confounded with times, making it difficult to distinguish plate effects from time effects. There are also formalin-fixed, paraffin-embedded samples among the TCGA RNA-seq samples, and these were excluded from the data discussed here. Low-quality samples and lowly expressed genes were also excluded from individual datasets before the analyses in this paper (Methods). The TCGA RNA-seq datasets are available in the form of raw gene counts, fragments per kilobase of transcript per million mapped reads (FPKM) and FPKM followed by upper-quartile normalization (FPKM.UQ).

### Library size, tumor purity and plate effects are major sources of unwanted variation across TCGA RNA-seq datasets

**Fig. 1:**
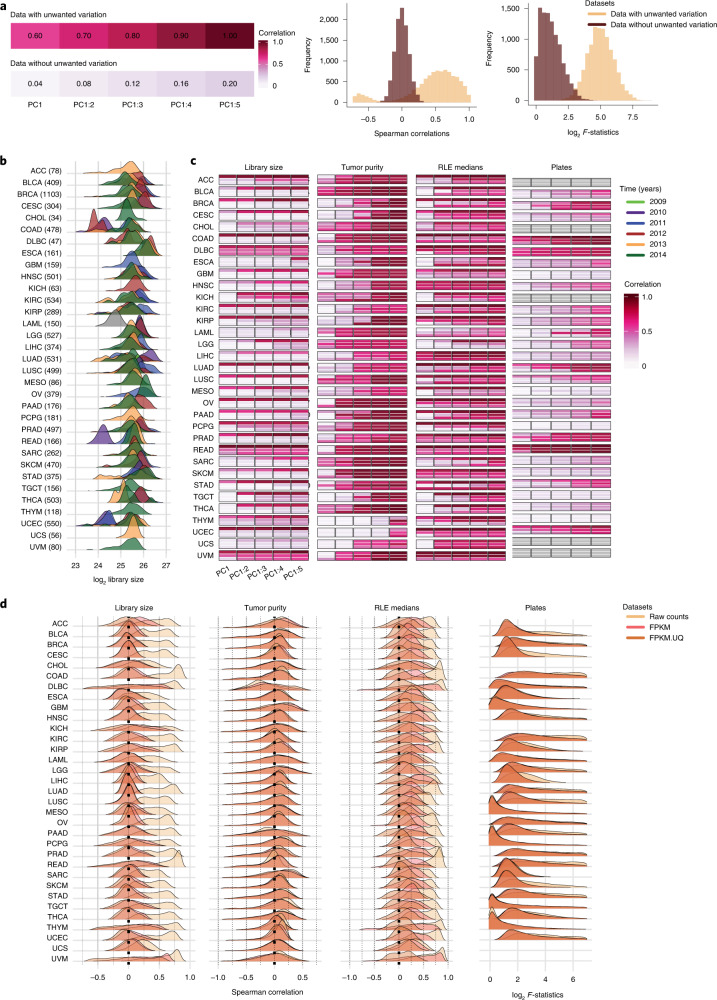
Unwanted variation in individual TCGA RNA-seq datasets. **a**, Illustrative examples showing data with and without unwanted variation. Data with unwanted variation exhibit high correlation between the first five PCs and this variation (top left). Data without unwanted variation have low correlation with unwanted variation (bottom left). The histograms show Spearman correlations and log_2_ *F-*statistics between individual genes and different sources of unwanted variation. Data with large library size and tumor purity variation show high Spearman correlations between individual gene expression and this variation. Data with plate effects exhibit high *F*-statistics obtained from ANOVA between individual gene expression and plates as factor. In contrast, data without such unwanted variation show low Spearman correlations and *F*-statistics. **b**, Distribution of (log_2_) library size colored by years for the individual TCGA cancer types. The year information was not available for the LAML RNA-seq study. The library sizes are calculated after removing lowly expressed genes for each cancer type. **c**, *R*^2^ obtained from linear regression between the first, first and second, and so on, cumulatively to the fifth PC and library size (first panel), tumor purity (second panel) and RLE medians (third panel) in the raw count, FPKM and FPKM.UQ normalized datasets. The fourth panel shows the vector correlation between the first five PCs cumulatively and plates in the datasets. Ideally, we should see no significant associations between PCs and sources of unwanted variation. Gray color indicates that samples were profiled across a single plate. **d**, Spearman correlation coefficients between individual gene expression levels and library size (first panel), tumor purity (second panel) and the RLE medians (third panel) in the datasets. The fourth panel shows log_2_ *F*-statistics obtained from ANOVA of gene expression levels by the factor: plate variable. Plates with fewer than three samples were excluded from the analyses. ANOVA was not possible for cancer types whose samples were profiled using a single plate.

We first considered the role of sample RNA-seq library size as a source of unwanted variation. Ideally, the gene-level counts should have no significant association with library size variation in a well-normalized dataset (Fig. 1a). Consequently, any downstream analysis, including dimensional reduction, gene co-expression and differential expression, should also not be influenced by library size variation.

For most TCGA RNA-seq studies, library sizes vary greatly both within and between years (Fig. 1b). The first five principal components (PC) cumulatively are strongly associated with (log) library size in the raw gene counts (Fig. 1c, first panel). The FPKM and FPKM.UQ normalizations reduced the effects of library size, but they showed shortcomings—high correlation between PCs and library size—in several cancer types (Fig. 1c, first panel). For each cancer type, the association between individual gene-level counts and library size was quantified using Spearman correlation (Fig. 1d, first panel, and Supplementary Fig. 2a). The results show that a large proportion of genes have high positive correlations with library size in the raw gene count datasets. However, in these datasets, there are reasonable numbers of genes whose expression levels have no correlation or a negative correlation with library size (Fig. 1d, first panel) and, thus, present a challenge for the standard RNA-seq normalizations. Supplementary Fig. 1 shows that the association between gene-level raw counts and library size is partially explained by average gene expression level and is never constant. The FPKM and FPKM.UQ normalizations introduce or exacerbate library size effects in genes whose expression has no or negative association with this variation. This will be discussed in more detail for the rectum adenocarcinoma (READ) and colon adenocarcinoma (COAD) RNA-seq datasets.

Next, we used linear regression and Spearman correlation analyses to quantify the variation in tumor purity in the TCGA RNA-seq datasets (Fig. 1c, second panel, and Fig. 1d, second panel). The results indicate the presence of substantial variation in tumor purity, and FPKM and FPKM.UQ normalizations cannot correct for this in the datasets (Fig. 1c, second panel, and Supplementary Fig. 2b). We discuss how the tumor purity variation can compromise downstream analyses, including gene co-expression and subtype identification, as was observed in the TCGA breast invasive carcinoma (BRCA) RNA-seq data.

In most TCGA RNA-seq studies, biospecimens were profiled necessarily across plates, which can impact on gene expression levels. Vector correlation and analysis of variance (ANOVA) (Methods) reveal the presence of plate effects in the raw gene counts, FPKM and FPKM.UQ normalized datasets (Fig. 1c, third panel). We found that the major known biological populations are well-distributed across plates in TCGA READ, COAD, lung adenocarcinoma and BRCA RNA-seq data, showing the absence of large confounding effects in the data.

Finally, we examined the medians of relative log expression (RLE)^23^ for the raw count and TCGA normalized datasets (Methods). In the absence of unwanted variation, the RLE medians should be centered around zero, so any deviation from zero indicates the presence of unwanted variation in the data. Supplementary Fig. 3 illustrates that the RLE medians of the raw count datasets deviate greatly from zero, which further confirms the presence of unwanted variation. We then investigated the associations between the first five PCs cumulatively and the RLE medians (Fig. 1c, third panel) and also computed the Spearman correlation between individual gene expression with the RLE medians for each cancer type (Fig. 1d, third panel). Ideally, we should see no associations; however, we see many associations in the raw counts and the FPKM and FPKM.UQ normalized datasets. We will demonstrate the importance of scrutinizing the association between the RLE medians and both principal component analysis (PCA) and individual gene expression in the TCGA breast cancer RNA-seq data.

Taken together, our results show that all the TCGA RNA-seq datasets, both raw and normalized, are greatly affected by the three major sources of unwanted variation. Next, we used the READ, COAD and BRCA RNA-seq datasets to illustrate the effects of unwanted variation on certain downstream analyses and show the performance and effectiveness of RUV-III with PRPS for these datasets. The details of each study are provided separately below.

### TCGA READ RNA-seq study

#### Study outline

The READ RNA-seq study involved 176 assays generated using 14 plates over 4 years. The RNA-seq library sizes vary greatly between samples profiled in 2010 and the other samples (Supplementary Fig. 4). The major gene-expression-based biological populations—consensus molecular subtypes (CMSs)^24^—were identified using the R package CMScaller^25^ (Methods) in the data normalized by different methods. See Supplementary Figs. 5 and 6 and the Supplementary File for further details. These subtypes will be used for both assessing the performance of normalization methods and creating PRPS for RUV-III normalization.

#### RUV-III removes substantial library size variation and plate effects from the data

**Fig. 2:**
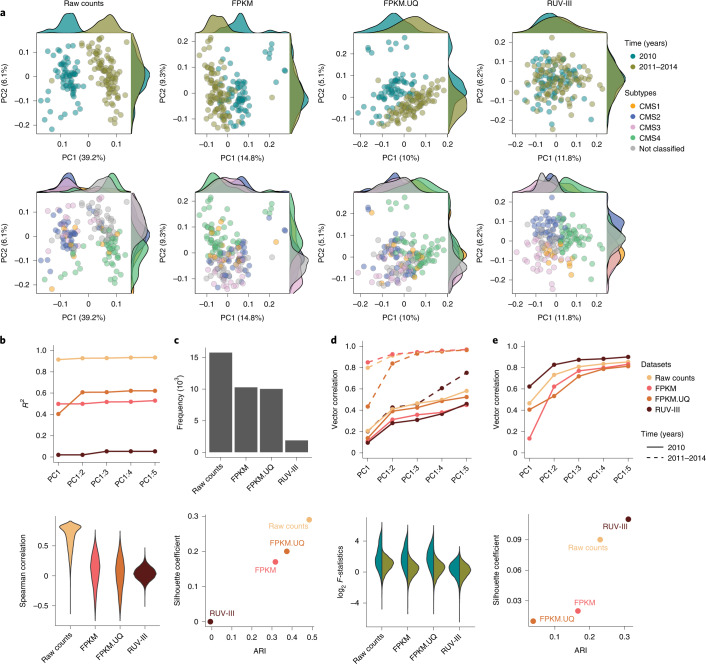
Performance assessment of different normalizations on the TCGA READ RNA-seq data. **a**, Top row: scatter plots of first two PCs for raw counts, FPKM, FPKM.UQ and RUV-III normalized data colored by key time intervals (2010 versus 2011–2014). Bottom row: same as the top row colored by the CMS. The CMSs were obtained for each dataset separately. **b**, Top: a plot showing the *R*^2^ of linear regression between library size and up to the first five PCs (taken cumulatively). Bottom: violin plots of Spearman correlation coefficients between the gene expression levels and library size for individual data. **c**, Top: the frequency of *P* < 0.05 obtained from DE analysis between samples with low and high library size. Bottom: Scatter plot shows silhouette coefficients and ARI for mixing samples from two different key time intervals. **d**, Top: a plot showing the vector correlation coefficient between plates and the first five PCs within each time intervals. Bottom: box plots of log_2_ *F*-statistics obtained from ANOVA within each key time interval for gene expression with plate as a factor. **e**, Top: a plot showing the vector correlation coefficient between CMS subtypes and up to the first five PCs. Bottom: a scatter plot displays silhouette coefficients and ARI for measuring the separation of CMS subtypes.

Substantial library size variation between samples profiled in 2010 and the other samples are clearly visible in the RLE and PCA plots (Supplementary Fig. 7a and Fig. 2a, top panel) of the raw count data. Although the FPKM and FPKM.UQ normalizations reduced this variation, both methods exhibited shortcomings—for example, by not fully mixing samples with large library size differences (Fig. 2a, top row).

PCA plots and linear regression between the first five PCs cumulatively and library size clearly illustrate that RUV-III with PRPS improved upon the FPKM and FPKM.UQ normalizations in removing the variation in library size from the data (Fig. 2a, top row, and Fig. 2b, top plot).

Spearman correlation analyses between the individual gene expression values and library size reveal a large proportion of genes showing strong positive or negative correlations with library size in the FPKM and FPKM.UQ normalized datasets, whereas this correlation was significantly reduced in the RUV-III normalized data (Fig. 2b, bottom). Furthermore, differential expression (DE) analysis (Methods) was performed between samples with high and low library size. Ideally, we should see little evidence of differential gene expression, whereas we see a lot in the FPKM and FPKM.UQ datasets, far more than in the RUV-III normalized data (Fig. 2c, top). Finally, the silhouette coefficient and adjusted Rand index (ARI) analyses (Methods) showed that RUV-III performs better in mixing the samples with large library size differences (Fig. 2c, bottom).

To examine plate effects and separate this variation from the large library size variation in the data, we performed our evaluation within each key time interval. The results showed that RUV-III clearly improves over the FPKM and FPKM.UQ normalizations in removing plate effects from the data (Fig. 2d).

Note that, here we have not attempted to remove variation caused by tumor purity. Consequently, the tumor purity estimates obtained from the RUV-III and FPKM.UQ normalized data were highly correlated (Supplementary Fig. 7b). This illustrates the ability of RUV-III to remove only the variation that the user wants to remove and no more—that is, to retain other variation that is of biological origin.

We next explored the relationship between RLE medians and both library size and tumor purity—the two major variations in the data—for the different normalizations (Supplementary Fig. 7c). The library size variation is the largest variation in the raw counts data, and RLE medians are strongly associated with this variation. The TCGA and RUV-III normalizations reduced the variation in the library size; therefore, the tumor purity became the largest variation in these datasets. Then, the RLE medians of the TCGA and RUV-III normalized data show a strong association with tumor purity (Supplementary Fig. 7d). These results were further supported by comparisons of the Spearman correlation analyses between the individual gene expression levels and RLE medians with the same analyses between the individual gene expression levels and library size and with tumor purity (Supplementary Fig. 8). Together, these results show the value of exploring the association of the RLE medians with known sources of unwanted variation in the data. Later, we will show that the RLE medians have no correlation with gene expression in the TCGA BRCA RUV-III normalized data when variations in both library size and tumor purity are removed.

#### RUV-III improves the separation between consensus molecular subtypes

Colorectal cancers are classified into four transcriptomic-based subtypes—CMSs—with distinct features^24^. PCA plots of the RUV-III normalized data show distinct clusters of the CMSs for the READ RNA-seq samples, whereas these subtypes are not as clearly separated in the TCGA normalized datasets (Fig. 2a, bottom row). To confirm the pattern of the CMS clusters in the PCA plots of the RUV-III normalized data, we applied PCA within the key time intervals in the FPKM and FPKM.UQ normalized datasets. The results show that the CMS clustering within each time interval in the FPKM.UQ data is highly consistent with that obtained with RUV-III using the full set of data (Supplementary Fig. 9).

Furthermore, the vector correlation analysis between the first five PCs cumulatively and the CMS confirmed that the RUV-III normalization leads to a better separation of the CMS clusters than the TCGA normalized datasets (Fig. 2e, top). These results were strengthened by silhouette coefficient and ARI analyses (Fig. 2e, bottom). Additionally, gene set enrichment analyses showed that the CMSs obtained from the RUV-III normalized data are associated with known gene signatures^25^ (Supplementary Fig. 6b). Supplementary Fig. 10 shows the Kaplan–Meier survival plots of the CMSs identified by different normalization methods. The survival outcome difference between CMS2 and CMS4 that were obtained from the RUV-III normalized data is clearer than the TCGA normalized datasets (Supplementary Fig. 10).

#### RUV-III improves gene co-expression and gene-level survival analyses

**Fig. 3:**
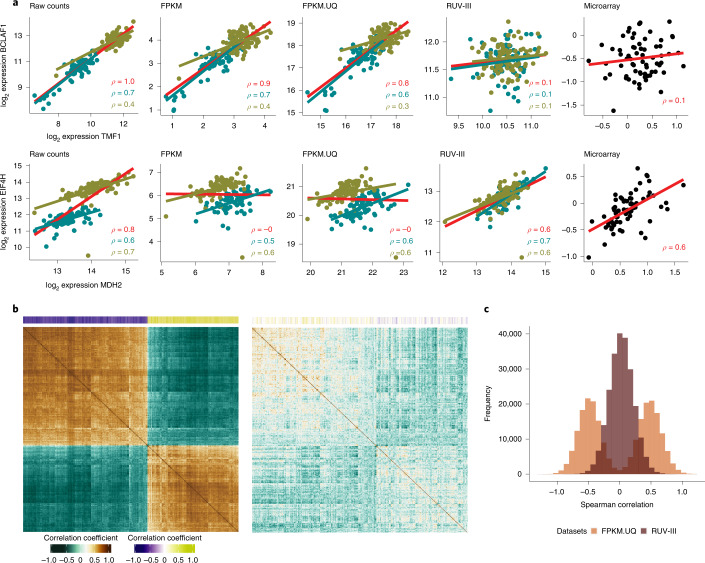
Gene co-expression analyses of TCGA READ RNA-seq data using different normalizations. **a**, First row: scatter plots of the gene expression levels of the *TMF1* and *BCLAF1* genes in the TCGA READ raw counts and differently normalized datasets. The red line shows overall association, and the cyan and olive lines show associations between the gene expression within 2010 samples and within the rest of the samples, respectively. Second row: same as the first row, for *MDH2* and *EIF4H* gene expression. **b**, The correlation matrix of expression levels of the genes with the 500 highest correlations with library size in the FPKM.UQ. The first plot is obtained using FPKM.UQ, and the second plot is obtained using the RUV-III normalized data. The colored bar along the top shows the correlation of individual genes with library size. The order of rows and columns is the same in both correlation matrices. **c**, Differences (*ρ*_microarray_ – *ρ*_RNA-seq_) of Spearman correlation coefficients for all possible gene–gene pairs calculated using the TCGA READ microarray and both the FPKM.UQ and RUV-III normalized RNA-seq data.

Unwanted variation introduced by the large sample library size differences can compromise downstream analyses, such as gene co-expression and gene-level survival analyses, in the TCGA READ RNA-seq data. This variation can have two effects on gene co-expression analysis. It can lead to apparent correlations between genes that are most likely un-correlated. For example, the correlation between the *TMF1* (TATA element modulatory factor 1) and *BCLAF1* (Bcl-2-associated transcription factor 1) genes are ρ = 0.8 and ρ = 0.7 in the TCGA FPKM and FPKM.UQ normalized data, respectively. The role of the *TMF1* gene has not been characterized in COAD, although the *BCLAF1* gene shows a pro-tumorigenic role in this cancer type^26^. One might suggest that the *TMF1* gene expression may have a role in tumorigenesis in colon cancer due to its high correlation with the *BCLAF1* gene expression. However, we see no such correlation in the RUV-III normalized data, which is consistent with the correlation obtained from an independent platform, namely the TCGA READ microarray data (Fig. 3a). On the other hand, the unwanted variation can obscure correlations between gene–gene expression levels that are likely to be truly correlated. For example, the overall correlation between the *MDH2* (malate dehydrogenase 2) and *EIF4H* (eukaryotic translation initiation factor 4H) genes is ρ = −0.05, whereas they exhibit a high correlation within each key time interval in the TCGA normalized data (Fig. 3a). The overall correlation of these genes was 0.7 in the RUV-III normalized data, consistent with what was seen in the TCGA READ microarray data (Fig. 3a). The *MDH2* and *EIF4H* genes show important roles in cancer growth and metastasis; thus, they are of clinical importance for cancer treatment^27,28^. The high correlation between these two genes revealed by RUV-III may suggest that they are involved in a co-expression network, which has not been previously reported.

We extended this analysis to all possible gene–gene correlations of the genes that have the highest correlation with library size in the FPKM.UQ normalized data (Fig. 3b). Strikingly, the results show numerous strong but likely spurious correlations between gene pairs in the FPKM.UQ normalized data, whereas using RUV-III significantly reduced these correlations (Fig. 3b).

Figure 3c depicts the differences (ρ_microarray_ – ρ_RNA-seq_) between all possible gene–gene Spearman correlations ρ using the TCGA READ microarray data and the FPKM.UQ and RUV-III normalized data.

**Fig. 4:**
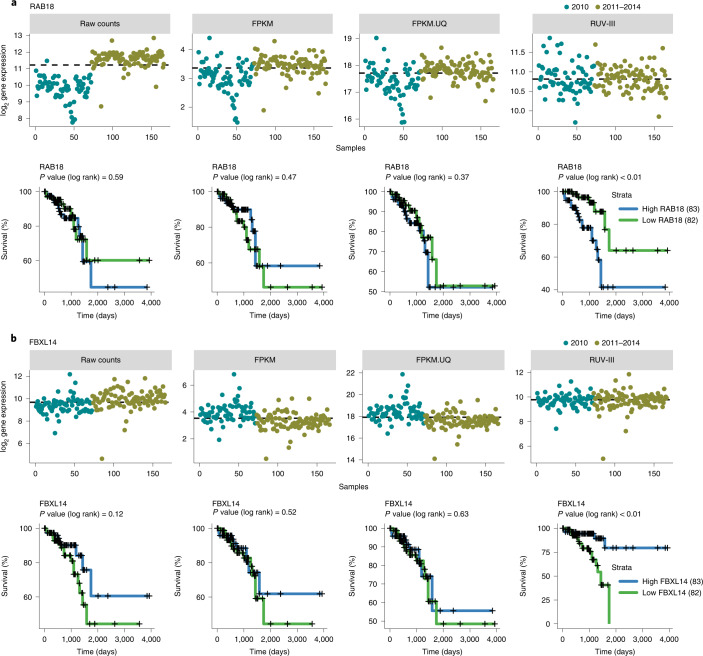
Association between gene expression and overall survival in differently normalized TCGA READ datasets. **a**, Upper part: plots of the expression levels of the *RAB18* gene across samples. The dashed lines represent the median expression level of the *RAB18 gene*. Lower part: Kaplan–Meier curves for samples with low (below median) and high (above median) expression of the *RAB18* gene. **b**, As in **a** for the *FBXL14* gene.

Association between gene expression and survival outcomes of patients is another downstream analysis that can be influenced by the library size variation in the TCGA READ RNA-seq data. For example, RUV-III, as opposed to the TCGA normalized data, revealed that the expression of the *RAB18* (Ras-related in brain 18) and *FBX14* (F-box and leucine-rich repeat protein 14) genes are highly associated with overall survival outcome of patients in the data (Fig. 4). The reason is clear from the expression patterns across time: dividing samples based on median expression mainly resulted in two groups with low and high library size, which was not biologically meaningful for the TCGA normalizations (Fig. 4). *RAB18* gene expression plays pivotal roles in cell proliferation and metastasis, and high expression is associated with poor survival in different cancer types^29^. *FBXL14* gene expression mediates the epithelial–mesenchymal transition (EMT) in cancer, which indicates that *FBXL14* could function as an EMT inhibitor to suppress metastasis in human cancers^30^. Other examples are *PTPN14* and *CSGALNACT2*, whose associations with survival have been previously shown in colorectal cancer (Supplementary Fig. 11)^31^. We found a remarkable number of genes whose expression levels were associated with survival using the RUV-III normalized data, which were not found using the FPKM and FPKM.UQ normalized data.

#### Gene-level counts are not proportional to library size

**Fig. 5:**
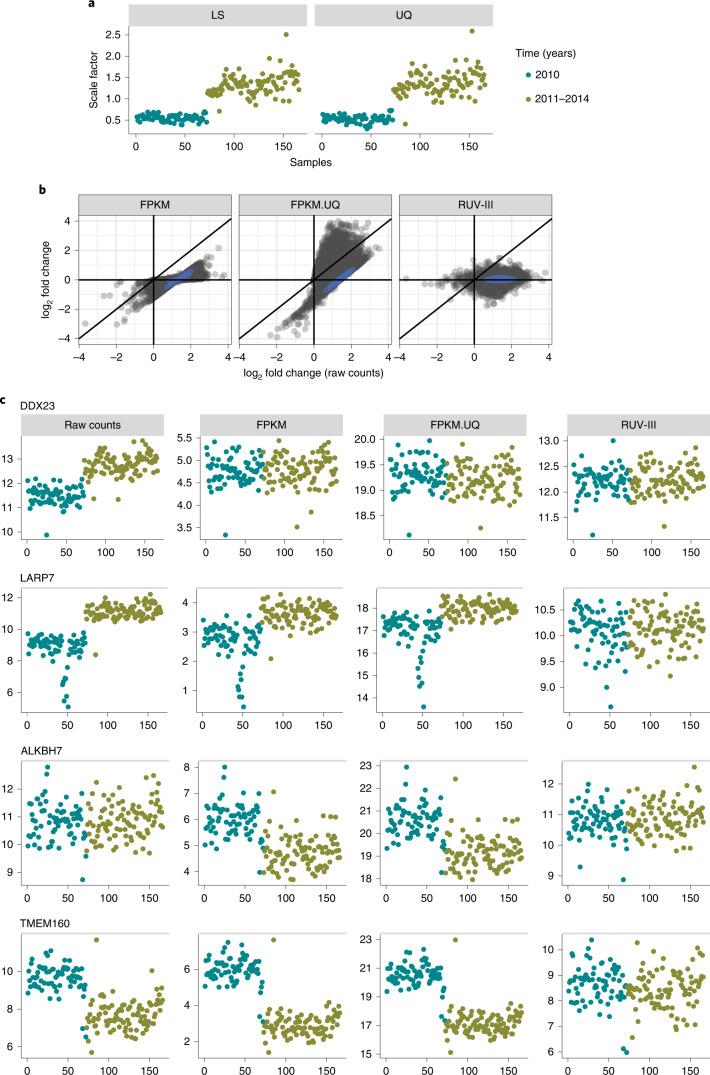
Relationship between gene-level (log_2_) counts and (log_2_) library size in the TCGA READ RNA-seq data. **a**, Global scale factors obtained by sample library sizes (LS) (left) and upper quartiles (UQ) (right) of READ raw counts versus time. **b**, Scatter plots of log_2_ fold change obtained from DE analyses of gene expression levels with the major time variation: 2010 versus 2011–2014; (log_2_) raw READ counts on the horizontal axes of all plots and differently normalized counts vertically. **c**, Expression patterns of four genes (*DDX23*, *LARP7*, *ALKBH7* and *TMEM160*) whose counts have different relationships with the global scaling factors calculated from the TCGA READ raw count data.

The FPKM and FPKM.UQ normalizations rely on global scale factors computed based on library size or upper quartiles of samples in the raw count data (Fig. 5a) to remove library size effects. These methods assume that gene-level counts all are proportional to the global scale factors. However, we show that, in the READ raw count data, different groups of genes exhibit different relationships to the global scale factors used in the FPKM and FPKM.UQ normalizations (Fig. 5b).

The first group consists of genes whose counts are proportional to the global scale factors. For these genes, the FPKM and FPKM.UQ normalizations are adequate to remove the association between library size variation and gene expression. The *DDX23* (DEAD-box helicase 23) gene is an example from this group (Fig. 5c, first row). The second group includes genes whose expression levels are greater than those expected using the global scaling factors, and so those factors are insufficient for adjusting their expression levels to be independent of library size. The *LARP7* (La ribonucleoprotein 7) gene represents the behavior of genes in this group (Fig. 5c, second row). The third group contains genes such as *ALKBH7* (AlkB homolog 7), whose expression levels are not associated with library size in the raw count data. Then, the FPKM and FPKM.UQ normalizations introduce the library size variation to the expression levels of genes in this group (Fig. 5c, third row). Finally, there are genes such as *TMEM160* (transmembrane protein 160) whose expression levels relate to library size in a manner opposite to that motivating the use of global scaling factors. Applying scaling factors to such genes exacerbates, rather than removes, variation associated with library size (Fig. 5c, fourth row).

Note that we found the same issue in the TCGA RNA-seq datasets, such as kidney chromophobe and uveal melanoma, where samples were profiled using a single plate (Fig. 1c, first panel, and Supplementary Fig. 12).

### TCGA COAD RNA-seq study

The COAD RNA-seq study involved 479 assays generated across 4 years. As with the READ RNA-seq data, there are large library size differences between samples profiled in 2010 and the other samples. The FPKM and FPKM.UQ normalizations removed library size effects from the data more effectively than was the case for the READ RNA-seq data, but these also had shortcomings.

It should be noted that the first two PCs of the FPKM and FPKM.UQ data did not reveal that the library size effects have not been properly removed. This highlights the importance of gene-level assessment, such as correlation between individual gene expression and library size or DE analysis between batches, to assess the performance of normalizations. See the Supplementary File and Supplementary Figs. 13–25 for full details of this dataset and results analogous to those just presented for the READ data.

### TCGA BRCA RNA-seq study

#### Study outline

The BRCA RNA-seq study involved 1,180 assays that were carried out on samples from 40 TSSs, distributed across 38 plates, and profiled over 5 years from 2010 to 2014 (Supplementary Fig. 26). The samples collected in 2010 and 2011 were profiled using one flow cell chemistry, and the remaining samples were profiled using a different flow cell chemistry (personal communication from TCGA). There were 94 adjacent normal breast tissue samples and seven paired primary-metastatic samples in the study (Supplementary Fig. 26). The major intrinsic biological populations, prediction analysis of microarray 50 (PAM50) of the TCGA BRCA RNA-seq samples, were identified using different approaches. See the Supplementary File and Supplementary Figs. 27 and 28 for full details.

#### RUV-III removes the effects of tumor purity, flow cell chemistries and library size

As with most of the other TCGA RNA-seq studies (Fig. 1), tumor purity is one of the major sources of variation in the BRCA study. For this dataset, we designed our PRPS to remove the effects of tumor purity as well as other technical variation (Methods).

**Fig. 6:**
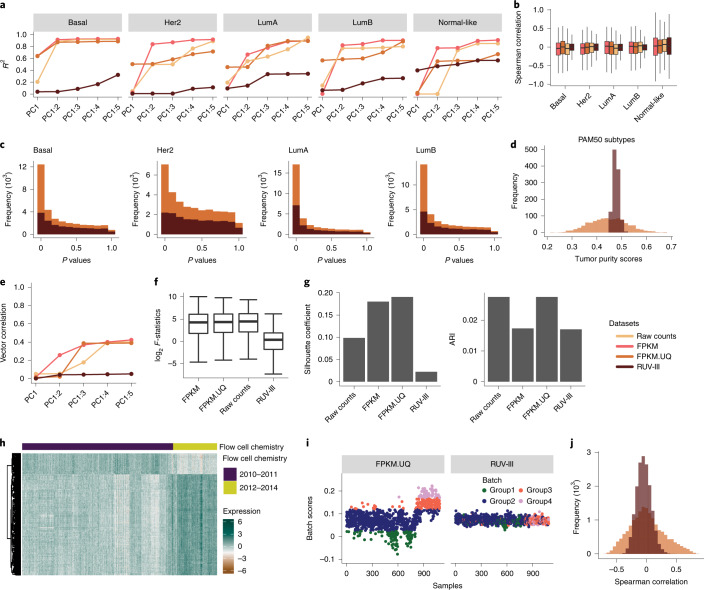
RUV-III removes tumor purity and flow cell chemistry variation from the TCGA BRCA RNA-seq data. **a**, *R*^2^ obtained from linear regression between the first five PCs (cumulatively) and tumor purity within individual PAM50 subtypes in the differently normalized datasets. The numbers of samples for each subtype and normalization are shown in Supplementary Fig. 27a. **b**, Box plots of Spearman correlation coefficients between individual gene expression and tumor purity levels in the differently normalized datasets (*n* = 16,537 genes). **c**, Unadjusted *P* value histograms of DE analysis between samples with low and high tumor purity within the four main PAM50 subtypes in the FPKM.UQ and the RUV-III normalized datasets. *P* values were obtained using Wilcoxon signed-rank test. **d**, Distributions of tumor purity scores in the FPKM.UQ and RUV-III normalized datasets. **e**, Vector correlation between the first five PCs (cumulatively) and flow cell chemistry in the normalized datasets. **f**, Box plots of log_2_ *F*-statistics obtained from ANOVA between individual gene expression levels and the flow cell chemistry factor in the differently normalized datasets (*n* = 16,537 genes). **g**, Bar charts of silhouette coefficients and ARIs showing the performance of different normalization methods in mixing samples from the two flow cell chemistries. **h**, Gene expression heat map of the 400 genes that are highly affected by the flow cell chemistries in the TCGA FPKM.UQ data (rows are clustered; columns are in chronological order of sample processing). **i**, Batch scores across samples in the FPKM.UQ (left) and RUV-III (right) normalized datasets. The batch scores were calculated by the singscore method using the 400 genes described in **h**. Samples were divided into four groups based on their batch scores. **j**, Spearman correlation coefficients between the batch scores and individual gene expression levels in the FPKM and RUV-III normalized datasets. In the box plots (**b** and **f**), the heavy middle line represents the median; the box shows the IQR; the upper and lower whiskers extend from the hinges no further than 1.5× IQR; and any outliers beyond the whiskers are shown as points.

Linear regression between the first five PCs cumulatively and tumor purity within the individual PAM50 subtypes showed that the RUV-III normalization substantially removed this variation from the data (Fig. 6a). These results were supported by Spearman correlation analyses between individual gene expression levels and tumor purity within each of the PAM50 subtypes and a DE analysis between samples with low and high tumor purity (Fig. 6b,c). The variation of tumor purity estimated using the RUV-III normalized data was significantly smaller than that observed in the corresponding measurements on the FPKM.UQ normalized data (Fig. 6d).

As mentioned above, the TCGA BRCA RNA-seq samples were profiled over two batches of flow cell chemistries. PCA plots of the FPKM and FPKM.UQ normalized datasets showed noticeable variation due to the use of two flow cell chemistries, whereas RUV-III effectively removed this variation from the data (Supplementary Fig. 29a). This conclusion was supported by a vector correlation analysis between the first ten PCs cumulatively and the binary flow cell chemistry variable, silhouette analyses, the ARI and ANOVA between individual gene expression measurements and the flow cell chemistry factor (Fig. 6e–g and Supplementary Fig. 29b,c).

An expression heat map of the most highly affected genes by the flow cell chemistries showed that different genes are affected in different ways (Fig. 6h). Interestingly, the heat map also revealed two clusters within the samples processed by the first flow cell chemistry. This suggests that there are additional sources of unwanted variation of unknown origin within each flow cell chemistry. To explore this more fully, we took the set of most highly affected genes by the flow cell chemistries and scored samples against this gene set (hereafter called the batch score) using the R/Bioconductor package singscore^32^ on the FPKM.UQ normalized dataset. Batch scores clearly distinguished samples from the flow cell chemistry batches and separated the samples into clusters within each flow cell chemistry (Fig. 6i). We then used cutoffs to divide the samples into four groups based on their batch scores. These groups were not visible in the batch scores obtained from the RUV-III normalized data (Fig. 6i). Spearman correlation analyses showed that a surprising number of genes had either high positive or high negative correlations with the batch scores in the FPKM.UQ normalized data (Fig. 6j), whereas these correlations were much lower in the RUV-III normalized data.

#### Tumor purity and flow cell chemistries effects compromise gene co-expression and survival analysis

**Fig. 7:**
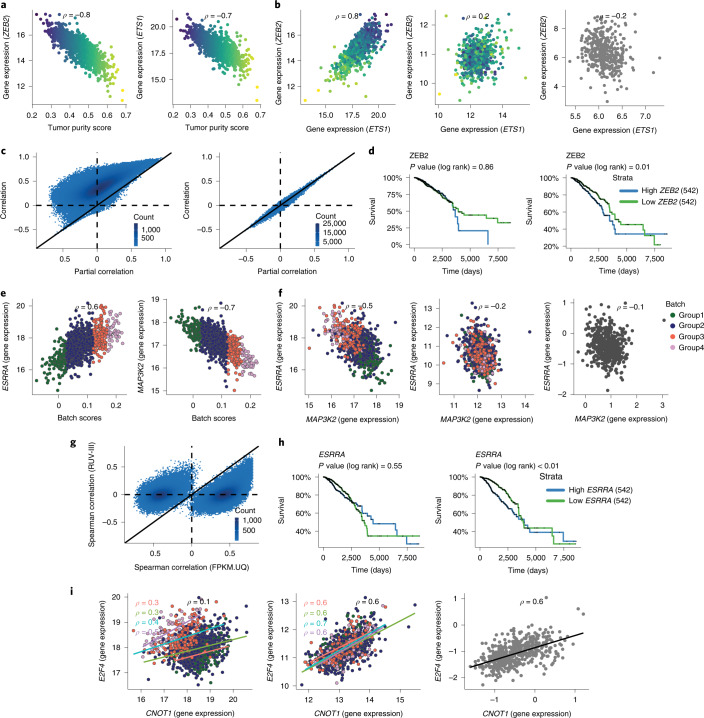
Impact of tumor purity and flow cell chemistry variation on gene co-expression and survival analysis in the TCGA BRCA RNA-seq data. **a**, Relationship between tumor purity scores and the *ZEB2* and *ETS1* gene expression in the FPKM data. **b**, Scatter plots exhibit relationship between the *ZEB2* and *ETS1* gene expression in the FPKM data (left), the RUV-III normalized data (middle) and the LCM microarray data (right). **c**, Scatter plots show the Spearman correlation coefficients and partial correlation coefficients for all possible pairs of the genes that have the 1,300 highest correlations with tumor purity in the TCGA FPKM.UQ (left) and RUV-III normalized data (right). **d**, Kaplan–Meier survival analysis shows the association between the *ZEB2* gene expression and overall survival in the FPKM.UQ (left) and the RUV-III normalized data (right). **e**, Relationship between the *ESSRA* and *MAP3K2* gene expression with the batch scores in the FPKM.UQ data. **f**, Scatter plots show the relationship between the *ESSRA* and *MAP3K2* gene expression in the FPKM.UQ (left), the RUV-III normalized data (middle) and the TCGA BRCA microarray data (right). **g**, Scatter plots display Spearman correlation coefficients of all possible pairs of genes that are highly affected by flow cell chemistries in the FPKM.UQ and the RUV-III normalized data. **h**, Kaplan–Meier survival analysis shows the association between the *ESSRA* gene expression and overall survival in the FPKM.UQ (left) and the RUV-III normalized data (right). **i**, Scatter plots exhibit the relationship between the *E2F4* and *CNOT1* gene expression in the FPKM.UQ (left), the RUV-III normalized data (middle) and the TCGA BRCA microarray data (right).

Just as we saw above with library size, tumor purity variation can affect downstream analyses, such as gene co-expression and the association between gene expression levels and survival outcomes of patients in the data. As with library size, this variation can introduce correlation between genes that are probably un-correlated. For example, Fig. 7a shows that the gene expression levels of *ZEB2* (zinc finger E-box-binding homeobox 2) and *ETS1* are both highly correlated with tumor purity. The *ZEB2* gene is a one of the regulators of the EMT process that induces invasion of cancer cells^33,34^. *ETS1* is member of a large family of transcription factors characterized by their ETS DNA‐binding domain. The gene appears to have dichotomous roles as an oncogene and a tumor suppressor gene in different cancer types^35,36^. The high correlation of *ETS1* with *ZEB2* in the TCGA BRCA RNA-seq data may confirm its oncogene role, but this is most likely a consequence of their correlations with tumor purity. The RUV-III normalized data and the breast cancer laser microdissection microarray data^37^ showed that the expression levels of these two genes are uncorrelated (Fig. 7b).

To extend this observation, we selected 1,300 genes whose gene expression levels are highly correlated with tumor purity and then calculated Spearman correlations between all possible pairs of these genes. In a matching analysis, we computed partial correlations between these pairs adjusting for tumor purity (Methods). Figure 7c shows that there are many gene pairs that have high correlations, but these are mostly likely a consequence of their correlation with tumor purity.

Variation in tumor purity can also affect the association between gene expression levels and survival outcomes. For example, the expression of the *ZEB2* gene shows to be associated with cancer progression and survival outcome in different cancer types^38,39^. The RUV-III normalization revealed that high expression of the *ZEB2* gene is associated with a poor outcome in the TCGA BRCA RNA-seq data, but this was obscured by variation in tumor purity in the FPKM.UQ normalized data.

Another example is the *STAB1* (stabilin 1) gene, whose expression levels are associated with survival in several cancer types, including breast cancer^40–42^. However, this association was only evident in the present data after removing variation in tumor purity. We found many more examples of such genes using the RUV-III normalized data.

The complex unwanted variation arising from the change in flow cell chemistry and the unknown source noted above clearly compromises estimates of gene co-expression in the FPKM.UQ normalized dataset. It introduces correlations between pairs of genes that are most likely not correlated. For example, the expression levels of the *ESRRA* (estrogen-related receptor alpha) and *MAP3K2* (mitogen-activated protein kinase kinase kinase 2) genes are positively correlated in this dataset; however, this correlation seems to be a consequence of the unwanted variation in the data (Fig. 7e), for we do not see it in either the RUV-III normalized data or the TCGA BRCA microarray data (Fig. 7f).

To extend this analysis, we first selected the genes that had the 1,000 highest correlations with the batch scores in the FPKM.UQ normalized data and calculated all gene–gene correlations between them in both the FPKN.UQ and RUV-III normalized datasets. Figure 7j shows that a large number of gene pairs have high correlations in the FPKM.UQ normalized data, something we do not see in the RUV-III normalized data.

Interestingly, the overall correlation between expression of the *E2F4* (E2F transcription factor 4) and *CNOT1* (CCR4-NOT transcription complex subunit 1) genes is *ρ* = *0.1*, and the average of the correlations of these genes within each of groups 1–4 of the unknown source of unwanted variation is *ρ* = *0.4* (Fig. 7i) in the FPKM.UQ normalized data. Both the RUV-III normalized and the TCGA microarray data show a high positive correlation between the expression levels of the *E2F4* and *CNOT1* genes.

Supplementary Fig. 30 shows that the RUV-III normalization removed library size effects from this dataset more effectively than was the case with the FPKM and FPKM.UQ normalizations.

#### RUV-III improves the separation of the PAM50 clusters

**Extended Data Fig. 1:**
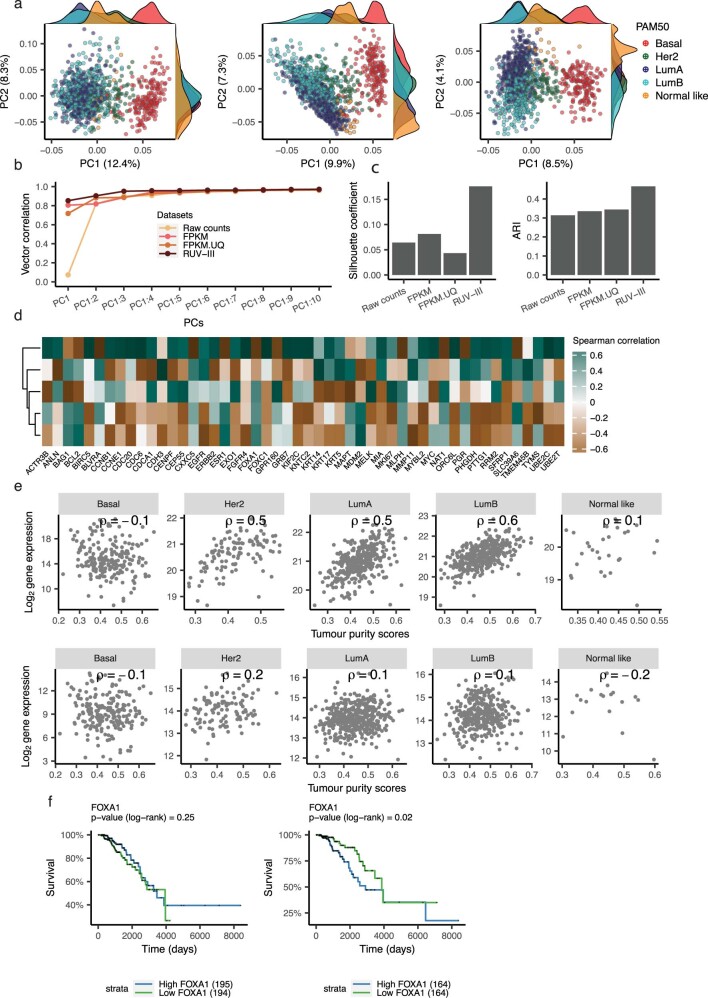
RUV-III improves the PAM50 clusters in the TCGA BRCA RNA-seq data. **a)** Scatter plot of the first two principal components colored by the PAM50 subtypes in the FPKM (left), FPKM.UQ (middle), and the RUV-III (right) normalized datasets. **b)** Vector correlation analysis between the first ten principal components cumulatively and the PAM50 subtypes in the differently normalized datasets. **c)** Silhouette coefficients and ARI showing how the PAM50 clusters are separated in the differently normalized datasets. **d)** The heatmap gives the Spearman correlation coefficients between the expression levels of the PAM50 signature genes and the tumor purity scores in the FPKM.UQ data. **e)** Scatter plots show relationship between the gene expression levels of FOXA1 and tumor purity within the individual PAM50 subtypes in the FPKM.UQ (first row) and the RUV-III normalized data (second row). **f)** Kaplan Meier survival analyses for samples with low and high expression of FOXA1 gene in Luminal-B subtype in the FPKM.UQ (left) and the RUV-III normalized data (right).

Breast cancer intrinsic subtypes, including HER2-enriched, basal-like, luminal A, luminal B and normal-like^43,44^, are based on a 50-gene expression signature (PAM50)^45^. PCA plots, vector correlation between the first ten PCs cumulatively and the PAM50 subtypes, silhouette coefficients and ARI (Extended Data Fig. 1a–c) all show that the RUV-III normalization led to better separation of PAM50 subtypes in the BRCA RNA-seq data. Kaplan–Meier survival analysis shows that the PAM50 calls obtained using RUV-III normalized data exhibit significant associations with overall survival outcomes of TCGA BRCA patients (Supplementary Fig. 27b,c).

It should be noted the PAM50 subtypes identified using the TCGA normalized datasets are compromised by tumor purity, particularly samples from normal-like subtype that show very low tumor purity. We applied the PAM50 classifier on the breast cancer laser capture microdissection (LCM) gene expression data and found no normal-like subtype in the dataset. The results confirm previous studies that show that the normal-like subtype is due to the low tumor purity of samples^46–48^.

Additionally, Spearman correlation analysis showed that several the PAM50 genes exhibit high correlation with tumor purity in the FPKM.UQ normalized data (Extended Data Fig. 1d). For example, expression of *FOXA1* (forkhead box A1) is highly associated with tumor purity in the Her2, luminal A and luminal B subtypes in the FPKM.UQ normalized data (Extended Data Fig. 1e). This observation suggests that variation in tumor purity might compromise the identification of PAM50 subtypes. In addition, this might also explain the differences between the PAM50 calls obtained from RUV-III normalized data, where the variation of tumor purity has been removed, and those obtained from the FPKM and FPKM.UQ normalized datasets (Supplementary Fig. 27a).

We explored the association between the expression levels of the PAM50 genes and survival within each of the PAM50 subtypes using both the FPKM.UQ and RUV-III normalized data. Interestingly, we found with the RUV-III normalized data that higher expression of the *FOXA1* gene is associated with poorer outcome in the luminal B subtype, a conclusion that was obscured by the variation in tumor purity of the TCGA RNA-seq data (Extended Data Fig. 1f).

### Normalization of multiple RNA-seq studies

We assessed the performance of RUV-III with PRPS on the normalization of multiple RNA-seq studies. In this analysis, we normalized three large breast cancer RNA-seq datasets, including TCGA and two cohorts from Brueffer et al. studies^49,50^. We did not have access to the raw counts data of Brueffer et al. studies, so we performed our normalization on the FPKM counts of all three studies. The lowly expressed genes were identified using the TCGA BRCA raw counts and removed from the other datasets. The PCA and RLE plots of the combined datasets show large variation between the TCGA and the other two studies (Supplementary Fig. 32a and 32b). As discussed above, we first need to identify sources of unwanted variation to create PRPS for RUV-III normalization. We used plates as batches for the TCGA BRCA RNA-seq data and the RLE medians (Methods) within each of the other two studies to identify batches. Their medians were clustered into three groups within each study. We performed PCA within each study using a set of RNA-seq housekeeping genes as negative control genes to explore the batches that were identified using the RLE medians. Supplementary Fig. 32c shows that the first and third PCs capture those batches. Then, the PAM50 subtypes were used as known major biological populations to produce five sets of PRPS (Supplementary Fig. 33a). The results demonstrated that RUV-III with PRPS leads to a satisfactory normalization by removing between-study and within-study variations and preserving the PAM50 clusters (Supplementary Fig. 33), whereas the other normalizations, quantile and upper quartile, show visible shortcomings. Furthermore, Supplementary Fig. 33d shows that several well-known gene–gene correlations^51^ have been preserved in the RUV-III normalized data. We also explored the correlation between the two pairs of genes, *CNOT1*_*E2F4* and *MAP3K2* _*ESRRA*, that were discussed in the TCGA BRCA RNA-seq data (Fig. 7). The true correlation between these two pairs of genes was preserved in the RUV-III normalized data (Supplementary Fig. 33d). The results demonstrate that RUV-III with PRPS is applicable to normalizing RNA-seq data from multiple studies. Note that we would have preferred to use RUV-III on the raw counts without any further normalization, but we were unable to do so here.

### Performance of RUV-III with poorly chosen PRPS

We evaluated the performance of RUV-III with poorly chosen PRPS on the TCGA READ and BRCA RNA-seq studies. To simulate poorly chosen PRPS, we randomly shuffled 20%, 40%, 60% and 80% of the biological labels, including the CMS and PAM50 subtypes, that were originally used to create PRPS for RUV-III normalization. The shuffling steps were repeated ten times for each proportion, and the results were averaged for normalization performance assessments.

The results show that, even with poorly chosen PRPS, RUV-III outperforms the FPKM and FPKM.UQ normalization in terms of removing large library size differences and preserving the CMS clusters in the TCGA READ RNA-seq data (Supplementary Fig. 34a,b). The correlations between two pairs of genes, *MDH2*_*EIF4H* and *TMF1*_*BCLAF1* (Fig. 3), were also preserved in the RUV-III datasets with poorly chosen PRPS (Supplementary Fig. 34c). Furthermore, the association between *RAB18* gene expression and the survival outcome (Fig. 4) was identified in all the RUV-III datasets with poorly chosen PRPS. However, we found this association for the *FBXL14* gene only in RUV-III with 20% shuffled labels (Supplementary Fig. 34d).

We performed a similar analysis on the TCGA BRCA RNA-seq data. Our results showed that the RUV-III normalizations with poorly chosen PRPS also show satisfactory performance compared to both FPKM and FPM.UQ in terms of removing the flow cell chemistry and tumor purity effects. However, RUV-III with 60% and 80% shuffled labels show a slightly lower performance compared to FPKM and FPKM.UQ normalization regarding the separation of the PAM50 subtypes (Supplementary Fig. 35). The gene–gene correlations and association between gene expression and survival outcomes demonstrated that the RUV-III normalizations with poorly chosen PRPS results in satisfactory normalization (Supplementary Fig. 35d–f).

Overall, our results illustrate that RUV-III shows a very satisfactory performance in a situation where PRPS is poorly chosen.

### Performance of RUV-III with partially known biological labels

We assessed the performance of RUV-III with PRPS in situations where the biological labels are partially known (hereafter called the RUV-III-P). To simulate such situations, we used one of the CMS subtypes, CMS4, to create PRPS for RUV-III normalization of the TCGA READ RNA-seq data. Note that this subtype is not present across all the plates. Our results clearly show that RUV-III-P normalization led to very satisfactory normalization by removing the large library size differences and plate effects and also preserving the CMS clusters (Supplementary Fig. 36). RUV-III-P also preserved the association between *RAB18* gene expression and survival outcomes in the TCGA READ RNA-seq data. However, this normalization did not show the same result for the *FXBL14* gene. This might be explained by the presence of the CMS4 subtype in eight out of 14 plates in the TCGA READ RNA-seq data.

Similar analyses were performed on the TCGA BRCA RNA-seq data. We used the basal and luminal A subtypes to create PRPS. The results demonstrated that performance of RUV-III-P was largely similar to the initial RUV-III normalization, in which all the PAM50 subtypes were used for producing PRPS (Supplementary Fig. 37).

These analyses show that RUV-III with PRPS can be used for normalization of RNA-seq data in situations where the biological labels of samples are only partially known.

## Discussion

The main goal of RNA-seq normalization is to remove unwanted variation that can compromise downstream analyses while preserving biological variation. A suitable normalization method for cancer RNA-seq data must be able to remove unwanted variation introduced by sample library size differences, tumor purity (where appropriate), batch effects and other technical variation in data.

We proposed an approach, called PRPS, to deploy RUV-III for normalization of RNA-seq in situations where suitable technical replicates are not available. Our PRPS approach requires the presence of at least a homogenous biological population across sources of unwanted variation. Then, we create pseudo-samples by averaging gene expression of a group of samples that are roughly homogeneous regarding the unwanted variation and biology. The gene expression differences between pseudo-samples are mainly unwanted variation. These samples will be used by RUV-III as a set of pseudo-replicates to estimate one aspect of unwanted variation in the data.

We made use of three TCGA RNA-seq studies to compare the performance of RUV-III with PRPS with the state-of-art normalizations proposed for RNA-seq data. RUV-III is not limited to TCGA data normalization, and we have also shown that the method can be used to normalize RNA-seq data, when the data come from multiple studies. Our comparisons are based on statistical summaries, biological positive and negative controls and concordance with the corresponding TCGA or independent microarray studies.

We began by carefully identifying different known sources of unwanted variation in all the TCGA RNA-seq raw count, FPKM and FPKM.UQ normalized datasets. We illustrated that library size, tumor purity and plate or time effects are major sources of unwanted variation in these studies, and we showed how they can influence downstream analyses. These unwanted variations are likely to affect other downstream analyses not investigated in this study.

In the TCGA READ RNA-seq study, noticeable library size differences between samples remained in the FPKM and FPKM.UQ normalized data due to the presence of genes whose raw counts showed weak or negative association with library size. In such situations, normalizations that rely on a global scale factor can introduce, rather than remove, library size variation. We found this issue in several TCGA cancer studies, even those that used a single plate for profiling. We took advantage of the gene-wise normalization ability of RUV-III to remove library size effects only from genes that are affected by this variation. Our results showed that RUV-III with PRPS effectively removed the library size effects from the TCGA READ RNA-seq data and led to better downstream analyses of gene–gene co-expression and association of gene expression with survival. Furthermore, the results showed that the variation due to tumor purity was highly similar for the TCGA and the RUV-III normalized datasets, as we did not attempt to remove the variation. This demonstrates the ability of RUV-III with PRPS approach to remove just the sources of unwanted variation that users aim to remove.

We found that the TCGA COAD RNA-seq data are affected by the same sources of unwanted variation that were identified in the corresponding READ RNA-seq data, although their effects were less severe in the COAD data compared to the READ data. The first two PCs of the FPKM and FPKM.UQ dataset did not show that the library size effects have not been properly removed. This highlights the importance of gene-level examinations to assess the performance of normalizations.

In the TCGA BRCA RNA-seq data, we designed our PRPS to remove variation in tumor purity as well as other sources of unwanted variation, including library size, flow cell chemistry and plate effects. We used LCM gene expression data to demonstrate the effects of tumor purity on the PAM50 subtype identification and gene co-expression analysis in the FPKM.UQ normalized data. We identified that the use of two flow cell chemistries introduced unwanted variation into the TCGA BRCA RNA-seq data. This introduced correlations between genes that were not truly associated and obscured the correlation between genes that were truly associated. We used the TCGA microarray data as an orthogonal platform to compare the gene expression patterns and their correlations in differently normalized datasets. The results of this comparison showed that the agreement between the RUV-III normalized data and the microarray data was much better than that found with the two TCGA normalized datasets.

The performance of RUV-III with the PRPS approach relies on the identification of major gene-expression-based biological populations in the data. Our results clearly showed that a rough identification of such populations using inadequate normalizations is satisfactory for creating the PRPS. In the three TCGA RNA-seq studies used in this study, the major biological populations were identified using TCGA normalized data that included sources of unwanted variation. However, we observed an equally good performance of RUV-III with PRPS using the biological populations identified in the RUV-III normalized data as we did using TCGA normalized data. Furthermore, we demonstrated that RUV-III is reasonably robust to poorly chosen PRPS.

Note that we could have created the PRPS using adjacent normal tissue, which is more homogeneous than cancer tissue, had these samples been more uniformly distributed across batches. However, we were not able to use such samples to create PRPS across the two flow cell chemistries for the BRCA study, as all 94 adjacent normal breast tissue samples were profiled using just one of the two chemistries. We found the same problem for the TCGA READ and COAD RNA-seq data. In these datasets, all normal adjacent tissues were profiled within a key time interval. It should be also noted that tumor purity is essentially the same across any set of technical replicates; thus, these sample are not useful to estimate and remove variation in tumor purity by the RUV-III normalization.

In large-scale genomics studies such as TCGA, samples are inevitably profiled using different reagents and platforms at different times, which can introduce unwanted variation into the data. As such, we strongly recommend including technical replicates across any possible source of unwanted variation. These samples can be used by any technical-based normalizations and considered as positive control to assess of any normalizations. We also recommend distributing the biology of interests across batches, to the extent that this is possible, as this will assist the use of RUV-III with PRPS. However, as it is difficult to predict all sources of unwanted variation and appropriately design technical replicates across them, RUV-III with PRPS provides a tool to remove this unwanted variation from large-scale cancer and genomics studies.

## Methods

### Datasets

The TCGA consortium aligned RNA-seq reads to the hg38 reference genome using STAR aligner and quantified the results at gene level using HTSeq and GENCODE version 22 gene annotation^52^. The TCGA RNA-seq data are publicly available in three formats: raw counts, FPKM and FPKM.UQ. All these formats for individual cancer types (33 cancer types, ~11,000 samples) were downloaded using the R/Bioconductor package TCGAbiolinks (version 2.16.1)^53^. The TCGA normalized microarray gene expression data were downloaded from the Broad GDAC Firehose repository (https://gdac.broadinstitute.org), data version 2016/01/28. TSSs and batches of sequencing plates were extracted from individual TCGA patient barcodes (https://docs.gdc.cancer.gov/Encyclopedia/pages/TCGA_Barcode/), and sample processing times were downloaded from the MD Anderson Cancer Center TCGA Batch Effects website: https://bioinformatics.mdanderson.org/public-software/tcga-batch-effects. Pathological features of patients with cancer were downloaded from the Broad GDAC Firehose repository (https://gdac.broadinstitute.org). The details of processing the TCGA BRCA RNA-seq samples using two flow cell chemistries were received by personal communication from Dr. Katherine Hoadley. The TCGA survival data reported by Liu et al.^54^ were used in this paper. The breast cancer LCM microarray dataset was downloaded using the GEOquery R/Bioconductor package (version 2.62.2) from the National Center of Biotechnology Information (NCBI) Gene Expression Omnibus (GSE78958 (ref. ^37^)). The two non-TCGA RNA-seq datasets were downloaded from the NCBI Gene Expression Omnibus with accession numbers GSE96058 and GSE81538 (refs. ^49,50^). The consensus measurement of purity estimation (CPE) was downloaded from the Aran et al. study^17^.

### Filtering samples and genes

We applied the following filtering steps to the individual TCGA RNA-seq datasets. Plates with fewer than three samples were removed. Samples with log_2_ library sizes of three median absolute deviations lower than the median of all log_2_ library sizes were excluded from the data.

The R/Bioconductor package biomaRt (version 2.48.3) was used to annotate genes. All pseudo-genes and immunoglobulin genes were excluded. For the pan-cancer analyses, we retained genes with at least 15 raw read counts in at least 20% of samples. We considered numbers of samples in the biological subpopulations and sources of unwanted variation when removing lowly expressed genes in the TCGA READ, COAD and BRCA data. To do so, we kept genes that have at least 15 counts in the smallest biological subpopulations within each of the key time intervals in the datasets.

### Tumor purity estimates

We estimated tumor purity for all TCGA RNA-seq cancer samples using the stromal and immune gene signatures (Supplementary Table 1) from the Yoshihara et al. study^55^ and the R/Bioconductor package singscore version (1.12.0)^32^. The stromal–immune scores were transformed to 1–stromal–immune scores for downstream analyses. These measurements are called tumor purity scores in this study. The tumor purity scores showed high positive correlation (mean = 0.95, Pearson correlation) with the ESTIMATE measurements from the Aran et al. study^17^ (Supplementary Fig. 38).

#### Sample library size

Sample library sizes were obtained by adding all gene raw counts for individual samples after removing pseudo-genes, immunoglobulin and lowly expressed genes. All sample library sizes are transformed to log_2_ in this study.

#### RUV-III normalization

Before we can describe the linear model underlying RUV-III, we need to introduce the *m* × *m*_1_ mapping matrix *M* connecting assays to distinct samples, which captures the pattern of replication in our assays. Here, *m* is the number of assays, and *m*_1_ is the number of distinct samples being assayed. $M\left( {i,h} \right) = 1$ if assay *i* is on sample *h* and $M\left( {i,h} \right) = 0$ otherwise. Each row of *M* sums to 1, and the columns sum to the distinct sample replication numbers, the elements of *M*^T^*M*. We also define an *m*_1_ × *p* design matrix *X* to capture the biological factor(s) of interest indexed by sample rather than assay. There are no constraints on *p*; indeed, *X* could be the *m*_1_ × *m*_1_ identity matrix. Our goal here is to remove unwanted variation, not to estimate regression parameters.

The linear model we use is:

$$Y = 1\mu + MX\beta + W\alpha + \varepsilon$$

where the data $Y = (y_{ij})$ and unobserved errors $\varepsilon = (\varepsilon _{ij})$ are *m* × *n*; the matrices *X* and *M* have just been defined; *µ* is the 1 × *n* row of gene means; *β is p* × *n*; the matrix *W* whose columns capture the unwanted variation is *m* × *k*; and *α* is *k* × *n*. 1 = 1_*m*_ is the *m* × 1 column vector of 1s. Here, *m* = number of assays, *n* = number of genes and *p* is the dimension of the wanted variation *X* and *k* that of the unwanted variation *W*. Assume that $W \bot 1$.

Also, we suppose that we have a subset of *n*_*c*_ of negative control genes whose *m* × *n*_c_ submatrix *Y*_c_ satisfies *Y*_c_ $= 1\mu _c + W\alpha _{\mathrm {c}} + \varepsilon _{\mathrm {c}}$, where we have assumed that *β*_c_ = 0—that is, that there is no true association between these genes and the biology of interest.

The projection $P_M = M(M^{{\mathrm {T}}}M)^{ - 1}M^{\mathrm {T}}$ replaces each entry *y*_*ij*_ of *Y* by the simple average of the entries *y*_*i*′*j*_ over all *i*′ for which $M\left( {i,h} \right) = M\left( {{i^\prime} ,h} \right) = 1$—that is, over all *i*′ such that *i*′ and *i* label replicate assays of the same unique sample (or pseudo-sample) labeled *h*.

Write *R*_*M*_ = *I* − *P*_*M*_ for the corresponding residual projector. This is our source of information on the unwanted variation that we will remove. If the replication is technical at some level, then *R*_*M*_*Y* mainly contains information about unwanted variation in the system after the technical replicates were created. Depending on the study details, technical replicates could be created immediately before the assay was run, in parallel with or immediately after sample was collected or somewhere in between. The earlier the creation of technical replicates, the more unwanted variation will be captured in their differences. The use of pseudo-replicates of suitable pseudo-samples enables us to start to deal with pre-technical unwanted variation.

Write the spectral decomposition of ${R_M}Y{Y^{\mathrm {T}}}R_M = UD{U^{\mathrm {T}}}$, where *U* is an *m* × *m* orthogonal matrix and *D* is an *m* × *n* diagonal matrix with entries ordered from largest to smallest eigenvalue. Let *P*_1_ be the orthogonal projection onto 1_*m*_.

For a chosen *k*, 1 ≤ *k* ≤ *m*−*m*_1_,

I. Define $\hat \alpha ^{(k)} = U^{\left( k \right)T}Y$, where *U*^(*k*)^ is the first *k* columns of *U*
II. Estimate *W* by regressing the centered negative controls (*I* − *P*_1_)*Y*_c_ on $\hat \alpha _{\mathrm {c}}^{(k){\mathrm {T}}}$
  $$\hat W^{\left( k \right)} = (I - P_1)Y_{\mathrm {c}}\left( {U^{\left( k \right){\mathrm {T}}}Y_{\mathrm {c}}} \right)^{\mathrm {T}}\left[ {\left( {U^{\left( k \right){\mathrm {T}}}Y_{\mathrm {c}}} \right)\left( {U^{\left( k \right){\mathrm {T}}}Y_{\mathrm {c}}} \right)^{\mathrm {T}}} \right]^{ - 1}$$
  Finally, we:
III. Form the adjusted/normalized *Y*, $Y^{(k)} = Y - \hat W^{\left( k \right)}$ $\hat \alpha ^{(k)}$.

#### PRPS

We used our recently developed normalization method, RUV-III, which makes essential use of technical replicates and negative control genes, to estimate unwanted variation and remove it from the data^8^. Ideally, technical replicates are placed across batches so that unwanted variation between any pair of batches is captured via differences of expression values between technical replicates. We previously showed the performance of RUV-III with pseudo-replicates in removing unwanted variation from gene expression data. Pseudo-replicates are samples from the same biological groups across batches. The idea of pseudo-replicates has also been used to remove batch effects in TCGA RNA-seq data with the unpublished algorithm EB++^56^.

As there are no technical replicates in the TCGA RNA-seq datasets, we developed an approach, PRPS, to be able to use RUV-III to remove unwanted variation from the data. To use RUV-III with PRPS, we first need to find sources of unwanted variation that we aim to remove from the data and identify relatively homogenous biological subpopulations among the samples. Then, we build pseudo-samples, which are in silico samples derived from a group of samples that are roughly homogeneous with respect to the unwanted variation and biology. The pseudo-samples from each biological groups will be a set of pseudo-replicates.

To make the process clear, we illustrate the PRPS for TCGA COAD and BRCA RNA-seq studies. For example, with the TCGA COAD samples, we regarded the 12 combinations of four CMSs (CMS1, CMS2, CMS3 and CMS4) with three microsatellite instability (MSI) statuses (MSI-high, MSI-low and MS-stable) as defining biological subpopulations. The key time intervals 2010 and 2011–2014 contribute the main unwanted variation that we saw after preliminary exploration of the data. As these times are totally confounded with sequencing plates (that is, different plates are used across different times), we considered plates to be the batches when defining PRPS. In addition, we were able to remove plate effects within each key time interval. As a result, to remove unwanted variation from the COAD data without removing biology, we created sets of pseudo-samples as follows: (1) select those biological subpopulations out of the 12 mentioned above that have at least three samples in at least two plates while also ensuring that, in the end, there are samples from plates within and across the two key intervals; and (2) average the gene expression levels of the corresponding samples within the individual plates to create one pseudo-sample. Having done this for all 12 biological subpopulations, we suppose all the pseudo-samples created across plates for a particular biological subpopulation to form a pseudo-replicate set.

For the BRCA data, we aimed to remove four different sources of unwanted variation—library size, tumor purity, flow cell chemistry and plate effects—from the data. Then, we needed to create distinct groups of PRPS for each source of unwanted variation. Note that we created a group of PRPS to remove the effects of plates and flow cell chemistries, as they are completely confounded with each other.

We considered the PAM50 subtypes (basal, Her2, luminal A, luminal B and normal-like) to define the major biological subpopulations and then created several sets of PRPS for each source of unwanted variation. To create PRPS for library size, we selected plates that have at least 12 samples of a particular PAM50 subtype and then selected the samples with the three highest and the samples with the three lowest values of library size. Then, we created two pseudo-samples within each PAM50 subtype per plate by averaging the gene expression values across each set of three high library size samples and each set of three low library size samples. We adopted the same approach explained above for the COAD data to create PRPS for plate effects. For removing the effect of tumor purity in the BRCA data, we defined sets of PRPS for each PAM50 subtype in addition to those that we created for removing library size, flow cell chemistries and plate-to-plate variation. We performed this by selecting the samples with the three highest and the samples with the three lowest values of tumor purity within each PAM50 subtype. Then, we created two pseudo-samples within each PAM50 subtype by averaging the gene expression values across each set of three high-purity samples and each set of three low-purity samples. Finally, the two pseudo-samples (average high and average low purity) created for each PAM50 subtype were regarded as forming a pseudo-replicate set—that is, a pair of pseudo-duplicates.

### Choice of negative control genes and *K*

Negative control genes for RUV-III are genes that are not highly affected by the biological factors of interest but are affected by one or more forms of unwanted variation in the data. We previously^8^ explained that our approach to negative controls is pragmatic: if regarding a set of genes as negative controls helps to remove unwanted variation using RUV-III, as evaluated by various metrics, then whether or not they are ideal negative control genes is not our concern. For the different cancer types discussed in this paper, we used different sets of negative control genes derived from either the literature (for example, housekeeping genes or genes found to be stable in the same, or a closely related, biological context) or the data itself (for example, genes found to exhibit little or no biological, but clear unwanted, variation). Candidate control genes have their effectiveness evaluated using various metrics after their use in RUV-III. It should be noted that unwanted variation mostly affects different subsets of genes in different ways.

To use RUV-III, a dimension *K* of unwanted variation needs to be determined. To find a suitable value, we repeated the analysis with a range of values of *K* and evaluated the quality of each analysis using different statistical metrics and prior biological knowledge. RUV-III is generally robust to overestimating *K* but not always.

### RUV-III normalization with PRPS for READ

As described above, the 11 combinations (we do not have CMS4_MSI-H) of the four CMS subtypes identified by the R package CMScaller on the READ FPKM and FPKM.UQ RNA-seq data (consensus calls were selected), and the three MSI statuses, were considered to be homogenous biological populations for the purpose of creating PRPS. Supplementary Fig. 39 displays the numbers of each 11 subpopulations within the individual plates. We created pseudo-samples from plates that have at least two samples of at least one of the 11 subpopulations. Supplementary Fig. 39b shows the library size of pseudo-samples within each subpopulation.

A set of negative control genes was selected in the following way. First, an ANOVA was carried out on FPKM.UQ normalized gene expression levels using the consensus calls of CMS subtypes as the factor, and the genes with lowest *F*-statistics were selected (~1,000 genes). PCA plots of the READ RNA-seq raw counts using the negative control genes showed that they capture the large library size differences between the key time intervals and do not capture CMS subtype differences (Supplementary Fig. 39c).

### RUV-III normalization with PRPS for COAD

Here we first defined CMS using the R package CMScaller on the COAD FPKM and FPKM.UQ RNA-seq data and selected the samples receiving the same CMS call for both (406 out of 479 samples). We used these CMSs and MSIs to define homogenous biological populations for the purpose of creating the PRPS (Supplementary Fig. 40). We used a slightly complicated approach to select a suitable set of negative control genes for the COAD study as follows: (1) carry out an ANOVA on the FPKM.UQ normalized gene expression values with CMS subtypes as the factor; (2) calculate Spearman correlations between FPKM.UQ normalized gene expression values and tumor purity; (3) calculate Spearman correlations between FPKM.UQ normalized gene expression values and the average expression level of a set of housekeeping genes^57^; and then (4) select genes (262 genes) that have lowest *F*-statistics (*F*-statistics < 20) from (1), the lowest correlations (ρ < 0.3) from (2) and the highest correlations (ρ > 0.9) from (3). PCA plots of the TCGA COAD RNA-seq raw count using negative control genes show that they capture the key time differences (Supplementary Fig. 40c).

### RUV-III normalization with PRPS for BRCA

The PAM50 subtypes were identified by using the R package genefu^58^ with the FPKM and FPKM.UQ normalized data. We selected samples with consensus PAM50 subtypes from the two datasets for creating PRPS. Three different groups of PRPS were then created to capture the library size, plate and flow cell chemistries and tumor purity effects (Supplementary Fig. 41).

The negative control genes were selected as follows: (1) carry out an ANOVA on the FPKM.UQ normalized gene expression values with PAM50 subtype as the factor, within each flow cell chemistry; (2) carry out a similar ANOVA with flow cell chemistry as the factor; (3) calculate Spearman correlations between FPKM.UQ normalized gene expression and purity values within the PAM50 subtypes; (4) calculate similar Spearman correlations with library size but with the raw counts; (5) select genes (4,500 genes) with the lowest *F*-statistics (*F*-statistics < 20) from (1), the highest *F*-statistics (*F*-statistics > 100) from (2), the highest correlations (|ρ| > 0.7) from (3) and the highest correlations (ρ > 0.07) from (4). PCA plots of the TCGA BRCA RNA-seq raw count using the negative control genes show that these genes capture all sources of unwanted variation in the data (Supplementary Fig. 42c).

#### Other RNA-seq normalization methods

We did not include the SVAseq^59^, ComBat-seq^22^ and RUVg^1^ methods in our analysis as these are not specifically designed for normalization, although they can be helpful for that task when the unwanted variation is orthogonal to the biology, something that is rarely known in advance. The same applies to the RUVs method provided in the RUVseq package^1^. Although if there are true replicates (missing from TCGA and most large cancer RNA-seq studies), it can be used to normalize RNA-seq datasets^5^.

### PCA

The PCs (in this context also called singular vectors) of the sample × transcript array of log counts are the linear combinations of the transcript measurements having the largest, second largest, third largest, etc., variation, standardized to be of unit length and orthogonal to the preceding components. Each will give a single value for each sample. In this paper, PCA plots are of the second PC values versus the first PC values and of the third PC versus the first PC. The calculations are done on mean-corrected transcript log counts, using the R code adopted from the R package EDAseq (version 2.26.1)^4^.

### RLE plots

RLE plots^23^ are used to reveal trends, temporal clustering and other non-random patterns resulting from unwanted variation in gene expression data. To generate RLE plots, we first formed the log ratio log(*y*_*ig*_/*y*_*g*_) of the raw count *y*_*ig*_ for gene *g* in the sample labeled *i* relative to the median value *y*_*g*_ of the counts for gene *g* taken across all samples. We then generated a box plot from all the log ratios for sample *i* and plotted all such box plots along a line, where *i* varies in a meaningful order, usually sample processing date. An ideal RLE plot should have its medians centered around zero, and its box widths and their interquartile ranges (IQRs) should be similar in magnitude. Because of their sensitivity to unwanted variation, we also examined the relationships between RLE medians with potential sources of unwanted variation and individual gene expression levels in the datasets. In the absence of any influence of unwanted variation in the data, we should see no such associations.

### Vector correlation

We used the Rozeboom squared vector correlation^60^ to quantify the strength of (linear) relationships between two sets of variables, such as the first *k* PCs (that is 1 ≤ *k* ≤ 10) and dummy variables representing time, batches, plates and biological variables. Not only does this quantity summarize the full set of canonical correlations, but it also reduces to the familiar *R*^*2*^ from multiple regression (see below) when one of the variable sets contains just one element.

### Linear regression

*R*^*2*^ values of fitted linear models are used to quantity the strength of the (linear) relationships between a single quantitative source of unwanted variation, such as sample (log) library size or tumor purity, and global sample summary statistics, such as the first *k* PCs (1 ≤ *k* ≤ 10). The lm() R function was used for this analysis.

### Partial correlation

Partial correlation is used to estimate Pearson (linear) correlation between two variables while controlling for one variables^61^. We computed the partial correlation between the expression levels of pairs of genes controlling for tumor purity using the pcor.test() function from the R package ppcor (version 1.1)^61^.

### ANOVA

ANOVA enables us to assess the effects of a given qualitative variable (which we call a factor) on gene expression measurements across any set of groups (labeled by the levels of the factor) under study. We use ANOVA *F-*statistics to summarize the effects of a qualitative source of unwanted variation (for example, batches) on the expression levels of individual genes, where genes having large *F*-statistics are deemed to be affected by the unwanted variation. We also use ANOVA tests (the aov() function in R) to assign *P* values to the association between tumor purity and molecular subtypes.

### *P* value histograms

It has been shown by Leek and Storey^62^ and others that histograms of the raw (that is, unadjusted) *P* values resulting from testing the same hypothesis (for example, of no differential expression across two or more groups of samples) on thousands of genes can be powerful indicator of the presence of unwanted variation. When there is no such variation and the underlying statistical model is appropriate, such *P* value histograms should be uniform apart from a possible peak near zero corresponding to genes where the null should be rejected. When there is unwanted variation, the histograms typically look very far from uniform apart from a peak near zero.

### Silhouette coefficient analysis

We used silhouette coefficient analysis to assess the separation of biological populations and batch effects. The silhouette function uses Euclidean distance to calculate both the similarity between one patient and the other patients in each cluster and the separation between patients in different clusters. A better normalization method will lead to higher and lower silhouette coefficients for biological and batch labels, respectively. The silhouette coefficients were computed using the function silhouette() from the R package cluster (version 2.1.2)^63^.

### ARI

The ARI^64^ is the corrected-for-chance version of the Rand index. The ARI measures the percentage of matches between two label lists. We used the ARI to assess the performance of normalization methods in terms of sample subtype separation and batch mixing. We first calculated PCs and used the first three PCs to perform ARI.

### DE analysis

DE analyses were performed using the Wilcoxon signed-rank test with log_2_-transformed raw counts and normalized data^65^. To evaluate the effects of the different sources of unwanted variation on the data, DE analyses were performed across batches. In the absence of any batch effects, the histogram of the resulting unadjusted *P* values should be uniformly distributed. The wilcox.test() R function was used for this analysis.

#### Identification of unwanted variation in TCGA RNA-seq datasets

**Extended Data Fig. 2:**
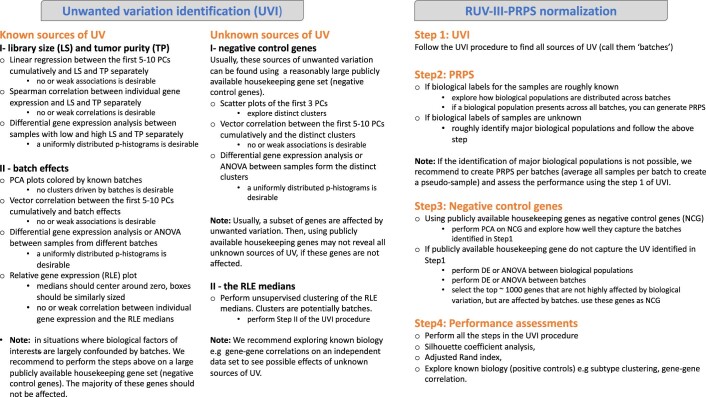
RUV-III with PRPS workflow. Workflow to identify known and unknown sources of unwanted variation, and apply RUV-III with PRPS normalization to RNA-seq data.

We made use of both global and gene-level approaches to identify and quantify unwanted variation in RNA-seq datasets (Extended Data Fig. 2). These approaches are also used to assess the performance of different normalization methods as removers of unwanted variation and preservers of biological variation in the data.

Our global approaches involve the use of PCA plots, linear regression, vector correlation analyses, silhouette coefficients, ARIs and RLE plots^23^. Our PCA plots (see above) are each of the first three PCs against each other, colored by known sources of unwanted variation—for example, time—or known biology—for example, cancer subtype. Linear regression is used to quantify the relationship between the first few PCs and continuous sources of unwanted variation, such as (log) library size. The *R*^2^ calculated from the linear regression analyses indicates how strongly the PCs capture unwanted variation in the data, and we perform these calculations cumulatively—that is, continuous source versus all of (PC_1_, …, PC_*k*_), for *k* = 1,…,5 or 10. Similarly to linear regression, we used vector correlation analysis to assess the effect on the data of discrete sources of unwanted variation, such as years or year intervals. Silhouette coefficients and ARIs were used to quantify how well experimental batches are mixed and known biology is separated. Finally, RLE plots^23^ were used to assess the performance of different normalizations in terms of removing unwanted variation from the data. We also explored the relationship between the medians and the IQRs of the RLE plots with sources of unwanted variation.

The gene-level approach includes DE analyses between experimental batches, looking at *P* value histograms and assessing the expression levels of negative control genes (see above), positive control genes (genes whose behavior we know), Spearman correlation and ANOVA between individual gene expression and sources of unwanted variation. These methods assess and quantify the effects of unwanted variation on individual gene expression levels in the RNA-seq datasets. See Methods section for more details about the assessment tools.

### Cancer subtype identification

We identified gene-expression-based cancer subtypes to create PRPS for RUV-III normalization. The CMScaller() function with default parameters from the R package CMScaller (version 2.0.1)^25^ was used to identify the CMSs in the TCGA READ and COAD RNA-seq data. The function provides classification based on pre-defined cancer-cell-intrinsic CMS templates.

We used two approaches to identify the PAM50 subtypes in the TCGA BRCA RNA-seq data. We implemented an algorithm proposed by Picornell et al.^66^ on the estrogen receptor (ER) balanced data. The ER estimates = 1.4 were selected to divide samples into ER-positive and ER-negative groups, and then the calibration (median normalization) factors were calculated.

In addition, we also used the molecular.subtyping() function with the PAM50 (single sample predictor) model from the R/Bioconductor package genefu (version 2.26.0) to identify the PAM50 subtypes. This method performs Spearman correlation between the expression of the PAM50 genes of each sample and PAM50 centroids (these data were downloaded here: https://github.com/bhklab/genefu) to calculate the correlation coefficient for individual PAM50 subtypes. Then, the individual sample is assigned to a particular PAM50 subtype based on its highest correlation coefficient.

We used Kaplan–Meier survival analysis to assess the prognostic values of different PAM50 identification approaches. The results showed that the PAM50 subtypes obtained by the genefu method are slightly more prognostic than those obtained by the other method (Supplementary Fig. 27).

### Spurious gene–gene correlation

We used two strategies to show spurious gene–gene correlations in the TCGA normalized data. First, we demonstrated how sources of unwanted variation, such as library size, tumor purity and batch effects, can introduce such correlations, which we did not see in the RUV-III normalized data. Second, we used the TCGA microarray gene expression data as orthogonal platform to explore and confirm these correlations. The TCGA microarray data contain gene expression data of subsets of samples that were profiled by RNA-seq platform. Our normalization assessment showed that the microarray data were not influenced by plates and time effects.

To explore spurious gene–gene correlations introduced by tumor purity in the TCGA data, we used the LCM microarray data, as these contain only gene expression signals from cancer cells. Note that we assessed both purity variation and quality of the LCM data.

### Reporting summary

Further information on research design is available in the Nature Research Reporting Summary linked to this article.

## Online content

Any methods, additional references, Nature Research reporting summaries, source data, extended data, supplementary information, acknowledgements, peer review information; details of author contributions and competing interests; and statements of data and code availability are available at 10.1038/s41587-022-01440-w.

## Supplementary information

Supplementary Information Supplementary Text and Figs. 1–42

Reporting Summary

Supplementary Table 1 Supplementary Table 1. Collated sample metadata for 11,023 TCGA RNA-seq samples, including date, plate identifier, center, tissue and purity

## Data Availability

The TCGA RNA-seq data are publicly available in three formats: raw counts, FPKM and FPKM.UQ. All these formats for individual cancer types (33 cancer types, ~11,000 samples) were downloaded using the R/Bioconductor package TCGAbiolinks (version 2.16.1). We have created summarized experiment objects containing expression data (raw counts, FPKM and FPKM.UQ), clinical and batch information and gene annotations for all the TCGA RNA-seq data. These files are deposited here: https://zenodo.org/record/6326542#.YlN56y8Rquo (ref. ^67^). The TCGA microarray gene expression data level 3 were downloaded from the Broad GDAC Firehose repository: https://gdac.broadinstitute.org, data version 2016/01/28. TCGA sample processing times were downloaded from the MD Anderson Cancer Center TCGA Batch Effects website: https://bioinformatics.mdanderson.org/public-software/tcga-batch-effects. The TCGA survival data were downloaded from the Liu et al. study^54^. The CPEs were downloaded from the Aran et al. study^17^. The breast cancer LCM and two non-TCGA RNA-seq datasets were downloaded from the NCBI Gene Expression Omnibus, with accession numbers GSE78958 (ref. ^37^), GSE96058 and GSE81538 (refs. ^49,50^) using the GEOquery R/Bioconductor package (version 2.62.2). The datasets that are required for the vignettes are deposited here: https://zenodo.org/record/6392171#.YlN6Yi8Rquo. The RUV-III normalized data of the TCGA READ, COAD and BRCA RNA-seq datasets are deposited here: https://zenodo.org/record/6459560#.YldJ4S8Rquo (ref. ^68^).

## Code availability

We developed an RShiny application and the tcgaCleaneR package to explore and remove unwanted variation in the TCGA RNA-seq datasets. All scripts were used to generate the main and supplementary figures, and two comprehensive vignettes that show all the steps in processing the TCGA READ and BRCA RNA-seq data are available on GitHub at: https://github.com/RMolania/TCGA_PanCancer_UnwantedVariation (ref. ^69^).
