# Supplementary material for: Removing unwanted variation from large-scale RNA sequencing data with PRPS
Source: Nat Biotechnol. 2022 Sep 15;41(1):82–95. doi: 10.1038/s41587-022-01440-w (PMC9849124; doi:10.1038/s41587-022-01440-w)
Supplement: Supplementary file 2 — Reporting Summary [file 41587_2022_1440_MOESM2_ESM.pdf]

nature research

Corresponding author(s): Ramyar Molania, Anthony T Papenfuss, Terence P Speed

Last updated by author(s): 18/06/2022

Reporting Summary

Nature Research wishes to improve the reproducibility of the work that we publish. This form provides structure for consistency and transparency in reporting. For further information on Nature Research policies, see our Editorial Policies and the Editorial Policy Checklist.

Statistics

For all statistical analyses, confirm that the following items are present in the figure legend, table legend, main text, or Methods section.

n/a	Confirmed	
☐	☒	The exact sample size (*n*) for each experimental group/condition, given as a discrete number and unit of measurement
☐	☒	A statement on whether measurements were taken from distinct samples or whether the same sample was measured repeatedly
☐	☒	The statistical test(s) used AND whether they are one- or two-sided<br>*Only common tests should be described solely by name; describe more complex techniques in the Methods section.*
☒	☐	A description of all covariates tested
☐	☒	A description of any assumptions or corrections, such as tests of normality and adjustment for multiple comparisons
☐	☒	A full description of the statistical parameters including central tendency (e.g. means) or other basic estimates (e.g. regression coefficient) AND variation (e.g. standard deviation) or associated estimates of uncertainty (e.g. confidence intervals)
☐	☒	For null hypothesis testing, the test statistic (e.g. *F*, *t*, *r*) with confidence intervals, effect sizes, degrees of freedom and *P* value noted<br>*Give P values as exact values whenever suitable.*
☒	☐	For Bayesian analysis, information on the choice of priors and Markov chain Monte Carlo settings
☒	☐	For hierarchical and complex designs, identification of the appropriate level for tests and full reporting of outcomes
☐	☒	Estimates of effect sizes (e.g. Cohen's *d*, Pearson's *r*), indicating how they were calculated

Our web collection on statistics for biologists contains articles on many of the points above.

Software and code

Policy information about availability of computer code

Data collection	NA
Data analysis	The R code of the RUV-III-PRPS method is available on this GitHub (https://github.com/RMolania/TCGA_PanCancer_UnwantedVariation). We also provided several vignettes to reproduce all the results and figures in the paper. The Rshiny and R packag apps are also available on the GitHub page. The other statistical analyses such as PCA, RLE, ANOVA, ... were performed using both built-in functions in R version 4.1.1 and publicly available R packages as follow. The R/Bioconductor packages: biomaRt (version 2.48.3), singscore version (1.12.0), EDAseq (version 2.26.1), genefu (version 2.26.0) and the R CRAN packages: ppcor (version 1.1), cluster (version 2.1.2) and CMScallerversion 2.0.1 were used. We described all the functions and packages in full details in the manuscript.

For manuscripts utilizing custom algorithms or software that are central to the research but not yet described in published literature, software must be made available to editors and reviewers. We strongly encourage code deposition in a community repository (e.g. GitHub). See the Nature Research guidelines for submitting code & software for further information.

Data

Policy information about availability of data

All manuscripts must include a data availability statement. This statement should provide the following information, where applicable:

- Accession codes, unique identifiers, or web links for publicly available datasets
- A list of figures that have associated raw data
- A description of any restrictions on data availability

The TCGA RNA-seq data are publicly available in three formats: raw counts, FPKM and FPKM with upper-quartile normalization (FPKM.UQ). All these formats for individual cancer types (33 cancer types, ~ 11,000 samples) were downloaded using the R/Bioconductor package TCGAbiolinks (version 2.16.1). We have created summarized experiment objects containing expression data (raw counts, FPKM and FPKM.UQ), clinical and batch information, and gene annotations for all the TCGA

RNA-Seq data. These files are deposited here (https://zenodo.org/record/6326542#.YlN56y8Rquo). The TCGA microarray gene expression data level 3 were downloaded from the Broad GDAC Firehose repository (https://gdac.broadinstitute.org), data version 2016/01/28. TCGA sample processing times were downloaded from the MD Anderson Cancer Centre TCGA Batch Effects website https://bioinformatics.mdanderson.org/public-software/tcga-batch-effects. The TCGA survival data were downloaded from Liu et al. study [54]. The consensus measurement of ¬¬purity estimation (CPE) were downloaded from the Aran et al. study [17].The breast cancer laser captured microdissected (LCM) and two none-TCGA RNA-seq datasets were downloaded from the NCBI Gene Expression Omnibus with accession numbers GSE78958 [37], GSE96058 and GSE81538 [49, 50] using the GEOquery R/Bioconductor package (version 2.62.2) from the NCBI Gene Expression Omnibus. The datasets that are required for the vignettes can be found here (https://zenodo.org/record/6392171#.YlN6Yi8Rquo). The RUV-III normalized data of the TCGA READ, COAD and BRCA RNA-Seq datasets are deposited (https://zenodo.org/record/6459560#.YldJ4S8Rquo).

Field-specific reporting

Please select the one below that is the best fit for your research. If you are not sure, read the appropriate sections before making your selection.

☒ Life sciences ☐ Behavioural & social sciences ☐ Ecological, evolutionary & environmental sciences

For a reference copy of the document with all sections, see nature.com/documents/nr-reporting-summary-flat.pdf

Life sciences study design

All studies must disclose on these points even when the disclosure is negative.

Sample size	We assessed the performance of our method on three large TCGA RNA-seq data and two other large breast cancer RNA-seq data. Individual dataset showed different forms of unwanted variation and complexity . Therefore, the datasets are sufficient to demonstrate the accurate performances of our method.
Data exclusions	Standard filtering were applied to remove lowly expressed genes and low quality samples. The full details can be found the method section.
Replication	All normalization methods were tested across multiple independent large-scale RNA-seq datasets.
Randomization	Our study did not involve allocating samples to experimental groups.
Blinding	Our study did not involve group allocation that requires blinding.

Reporting for specific materials, systems and methods

We require information from authors about some types of materials, experimental systems and methods used in many studies. Here, indicate whether each material, system or method listed is relevant to your study. If you are not sure if a list item applies to your research, read the appropriate section before selecting a response.

Materials & experimental systems

n/a	Involved in the study	
☒	☐	Antibodies
☒	☐	Eukaryotic cell lines
☒	☐	Palaeontology and archaeology
☒	☐	Animals and other organisms
☒	☐	Human research participants
☒	☐	Clinical data
☒	☐	Dual use research of concern

Methods

n/a	Involved in the study	
☒	☐	ChIP-seq
☒	☐	Flow cytometry
☒	☐	MRI-based neuroimaging
